# Supplementary material for: MetaRibo-Seq measures translation in microbiomes
Source: Nat Commun. 2020 Jun 29;11:3268. doi: 10.1038/s41467-020-17081-z (PMC7324362; doi:10.1038/s41467-020-17081-z)
Supplement: Supplementary file 10 — Supplementary Data 7 [file 41467_2020_17081_MOESM10_ESM.zip › File2/Confidence_High_Taxonomy/225163_out.krona.html]

Javascript must be enabled to view this page.

members
magnitude
magnitudeUnassigned
count
unassigned
taxon
rank

225163\_out

2

superkingdom
2
2

1239
phylum
2

2
909932
class

1843489
order
2

family
31977
2

1
2
29465

SRS104316\_contig\_number\_contig-100\_7114.7115
genus

39777
species
1

SRS016133\_contig\_number\_16238
